# A Practical Treatise on the Diseases of the Testis and of the Spermatic Cord and Scrotum, with Illustrations

**Published:** 1843-10-28

**Authors:** 


### BIBLIOGRAPHICAL NOTICE.

*A Practical Treatise on the Diseases of the Testis and of the Spermatic Cord and Scrotum, with Illustrations.* By T. B. Curling, Lecturer on Surgery, &c. Edited by P. B. Goddard, M. D., M. A. P. S., M. A. N. S. &c. Philadelphia, Carey & Hart. 1843. 8vo. pp. 568.

No monograph was more needed by the medical profession than one on the Diseases of the Testis. The noble work of the late Sir Astley Cooper on this subject is, from its very nature and high price, beyond the reach of students and most surgical practitioners. It is a fine monument of talent, industry and observation; but it wants that conciseness and practical character, essential to popularity.

The present work is a very complete manual on a most important class of diseases, derived from many years of careful study and investigation, and a thorough practical knowledge supplied by abundant opportunities. Our author has, in general, availed himself fully and judiciously of the labors of his predecessors; we regret that he has not consulted more frequently the contributions of the present French Surgeons to the affections of this organ, more especially those of Velpeau, Berard, Vidal, Ricord, &c.; this omission is particularly glaring in the chapter on "Tubercular Disease of the Testis."

The first part is occupied with the Anatomy of the Testis. The account of the structure of the Gubernaculum and the descent of the testis is original with Mr. Curling, and is now generally admitted by anatomists. Part second treats of the "Diseases of the Testis," arranged under the following heads.

I. Congenital Imperfections and Malformations. II. Atrophy of the Testis. III. Injuries of the Testis. IV. Hydrocele. V. Hematocele. VI. Orchitis. VII. Tubercular Disease of the Testis. VIII. Carcinoma of the Testis. IX. Cystic Disease of the Testis. X. Fibrous Transformation of the Testis. XI. Ossific Deposits in the Testis. XII. Loose Bodies in the Tunica Vaginalis. XIII. Spermatocele. XIV. Fœtal Remains in the Testis. XV. Entozoa in the Testis. XVI. Nervous Affections of the Testis. XVII. Sympathetic and Functional Disorders of the Testis. XVIII. Castration.

Part Third includes "Diseases of the Spermatic Cord." I. Varicolele. II. Adipose Tumors of the Cord. III. Spasm of the Cremaster Muscle.

"Diseases of the Scrotum" occupy the Third and concluding Part. I. Injuries of the Scrotum. II. Prurigo Scroti. III. Varicose Veins of the Scrotum. IV. Pneumatocele. V. Œdema Scroti. VI. Diffuse Inflammation of the Scrotum. VII. Mortification of the Scrotum. VIII. Elephantiasis Scroti. IX. Hypertrophy of the Scrotum. X. Cancer Scroti. XI. Melanosis of the Scrotum. XII. Adipose Tumors of the Scrotum. XIII. Fibrous Tumors of the Scrotum. An Appendix is added containing the recent discovery by Mr. Liston of Spermatozoa in the fluid of an encysted Hydrocele of the testis, with the author's observations and explanation of the occurrence of these bodies in this case.

From this summary of the contents of Mr. Curling's work an idea of its comprehensiveness will be obtained. When we say that the execution, with very few exceptions, corresponds with the conception, a very imperfect idea of its merits will be given. In a future number we shall present our readers with an analytical and critical notice. In the mean time we must express the great satisfaction that we have derived from its perusal, and urge upon all those who are anxious to possess a valuable book, its immediate purchase.

The American Editor, Dr. Goddard, has carefully revised the present edition, and made several additions of value. We cannot close our notice, without mentioning the typography of the volume, and the excellence of the Wood-Cuts, which are by Gilbert, many of them surpassing any of his previous efforts. The book is admirably printed, on thick white paper, with beautiful type, and dark ink, and complete justice has been done in striking off the cuts. We can conscientiously say that it is the best printed medical work that has issued from the press of this country, and quite equal in many respects to the finest English editions. For this the profession is indebted to the liberality of the publishers.

